# Multiscale method for modeling binding phenomena involving large objects: application to kinesin motor domains motion along microtubules

**DOI:** 10.1038/srep23249

**Published:** 2016-03-18

**Authors:** Lin Li, Joshua Alper, Emil Alexov

**Affiliations:** 1 Department of Physics, Clemson University, Clemson, SC 29634, USA

## Abstract

Many biological phenomena involve the binding of proteins to a large object. Because the electrostatic forces that guide binding act over large distances, truncating the size of the system to facilitate computational modeling frequently yields inaccurate results. Our multiscale approach implements a computational focusing method that permits computation of large systems without truncating the electrostatic potential and achieves the high resolution required for modeling macromolecular interactions, all while keeping the computational time reasonable. We tested our approach on the motility of various kinesin motor domains. We found that electrostatics help guide kinesins as they walk: N-kinesins towards the plus-end, and C-kinesins towards the minus-end of microtubules. Our methodology enables computation in similar, large systems including protein binding to DNA, viruses, and membranes.

Many biological processes involve the association of a single protein ligand to a larger protein complex receptor. An antibody binds to a virus^1^; a G protein binds to a G protein coupled receptor (GPCR)^2^; and a kinesin walks on a microtubule^3^,^4^, for example. At the molecular and atomic level, these processes are governed by the interplay of various forces, among which electrostatic force dominates the events before physical docking.

Molecular modeling techniques have enabled the study of ligand-receptor binding energetics^5^. The most accurate molecular modeling techniques involve explicitly representing and calculating the interactions between every atom in the system, including the proteins, solvent and soluble ions. For relatively small systems this is a manageable problem^6^, but for very large systems the computational cost is forbiddingly high^7^. To simplify the computations, multiple molecular modeling methods incorporate implicit expressions for the solvent, and they calculate the electrostatic energies, representing the longest-range interactions in biomolecular systems, using Poisson-Boltzmann equation^8^. However, many systems are still too large and complex to be fully modeled, even with software using implicit solvents, forcing either a simplification or truncation of the system that may lead to the loss of potentially important interactions^9^ or an extremely computationally intensive scheme^10^,^11^.

A promising approach for dealing with the complexity of systems with large dimensions is to apply different levels of resolution, from coarse-grained to atomistic details, in the modeling. In parallel, one can combine various biophysical and geometrical characteristics to deliver the quantity of interest. Such an approach is typically referred to as multiscale modeling^12^,^13^,^14^,^15^. The multiscale method was used to model cardiac action potential^16^, elastic properties of microtubules^17^,^18^, microtubule stability^19^, virus capsid dynamics^20^, geometrical modeling of macromolecules^15^, molecular dynamics modeling^21^ and many others^14^,^22^,^23^.

Here we developed a new multiscale approach to modeling large protein-protein binding systems in a computationally efficient way. Because calculating the electrostatic potential of biological molecules and assemblages is the most complex and intensive computational task^24^,^25^,^26^,^27^,^28^,^29^,^30^,^31^, it represents the bottleneck in performing computations on large systems. Our multiscale approach specifically targets efficiency improvements in the electrostatic energy calculations. We developed a novel algorithm that first calculates electrostatic energy at a course-grained resolution in the entire system. Then it transfers the information from the entire environment to a focused local region of interest and calculates the electrostatic energy at a significantly finer resolution.

To find ligand binding pathways, we first map the binding energy landscape at pre-determined rigid-body translations and rotations of the ligand with respect to the receptor using the computational focusing algorithm. We place the resulting energies in a lookup table. Then, we calculate multiple physically permissible binding pathways using a Monte Carlo simulation and the Metropolis algorithm. This Markov chain procedure ensures that the pathway satisfies appropriate Boltzmann probabilities calculated from the binding energy lookup table. Finally, we evaluate the results by quantifying the ensemble behavior of many calculated pathways.

We demonstrate the advantages of this methodology with an investigation of kinesin motor domain binding to a microtubule. Kinesins are processive microtubule motors that are critical to cellular processes including mitosis, meiosis, microtubule dynamics regulation, and intracellular transport^3^,^4^,^32^,^33^,^34^,^35^,^36^,^37^,^38^,^39^,^40^. Conformational changes of the motor induced by the hydrolysis of ATP and binding of the microtubule drive the hand-over-hand directional motility of kinesin^41^,^42^,^43^,^44^. At the same time, electrostatic interactions between charged residues on the microtubule and the kinesin motor domain are important to maintaining the directional bias kinesins^45^. However, the role of electrostatics in the molecular mechanism of kinesin’s motion is not fully understood despite multiple experimental^41^,^42^,^43^,^44^ and computational^42^,^45^ studies because the size of the microtubule-kinesin system makes computational modeling difficult^46^,^47^. Using our computational focusing method, we overcame these modeling difficulties. We computationally investigated of the role of electrostatics in kinesin’s directional motion along a complete microtubule accounting for all long-range electrostatic effects. Specifically, we investigated the differences between N-kinesins that move toward the plus-end of microtubules and C-kinesins that move toward the minus-end of microtubules. Our results demonstrate that the method is useful for investigating macromolecular binding in variety large systems, including protein-protein, protein-DNA, protein-virus, protein-membrane complex, and protein-nanoparticle binding.

## Results

### Computational focusing

We developed a computational focusing method to accelerate the electrostatic energy calculations in molecular modeling studies of large protein-protein and protein-nanoparticle binding systems. To adequately account for all long-range electrostatic contributions to the binding energy of proteins to large complexes, molecular modeling software, such as DelPhi^48^,^49^, must perform computations on structures that are as large as 1000^3^ Å^3^. Based on a resolution of 2 grids/ Å, this yields a three dimensional mesh with 8 × 10^9^ grid points, which requires more than 100 GB of RAM and a long time to complete the calculations. We exploit the opportunity for focused, local refinement in the computation by identifying a small area of interest within the entire system.

**Figure 1:**
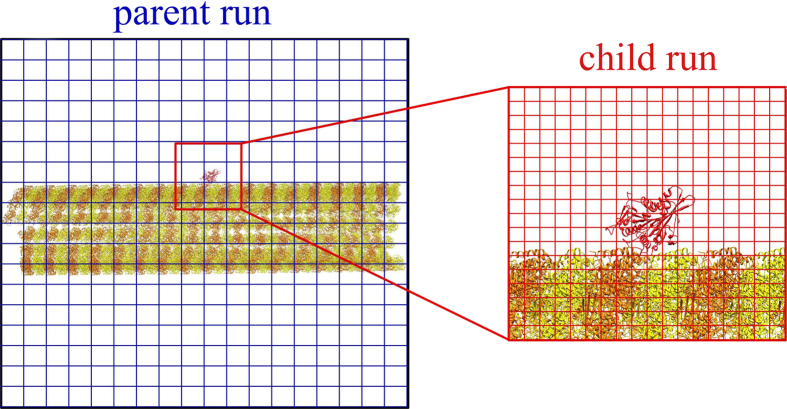
The focusing method. (left) The entire system and a representative (not to scale) grid indicating the low-resolution nature of the electrostatic calculations done in a parent run. (right) The area of interest and a representative grid indicating the high-resolution nature of the electrostatic calculations done in the child run.

Our focusing method greatly reduced computation time using parent-child runs. In the parent run, DelPhi calculates and saves the electrostatic potential of the entire system at low resolution. In subsequent child runs, DelPhi calculates the electrostatic potential in the area of interest at higher resolution using the electrostatic potential calculated in the parent run as a boundary condition on the area of interest (Fig. 1 Methods section for more detail). The computational focusing method allows the boundary of child runs to be located anywhere within the parent run and to have any resolution. It significantly saves calculation time because only the area of interest is calculated at high resolution and multiple child runs share the same parent run. Additional benefit can be achieved by nesting the focusing method at multiple levels yielding grandparent-parent-child runs, for example.

We calculated binding pathways by performing Monte Carlo simulations (see Methods section for details). The ligand begins its pathway at a user defined initial position. In each time step, the Monte Carlo simulation randomly selects a neighboring position pre-generated by the rigid-body sampling module for the ligand by performing a three-dimensional translation and rotation. We applied the Metropolis algorithm, which uses the difference in binding energy pre-calculated at every possible location using the energy module and the computational focusing method, to determine if the neighboring position is accepted or not. By stringing together the accepted positions, we generated a binding pathway, indicating positions and orientations where the ligand can be found.

**Figure 2:**
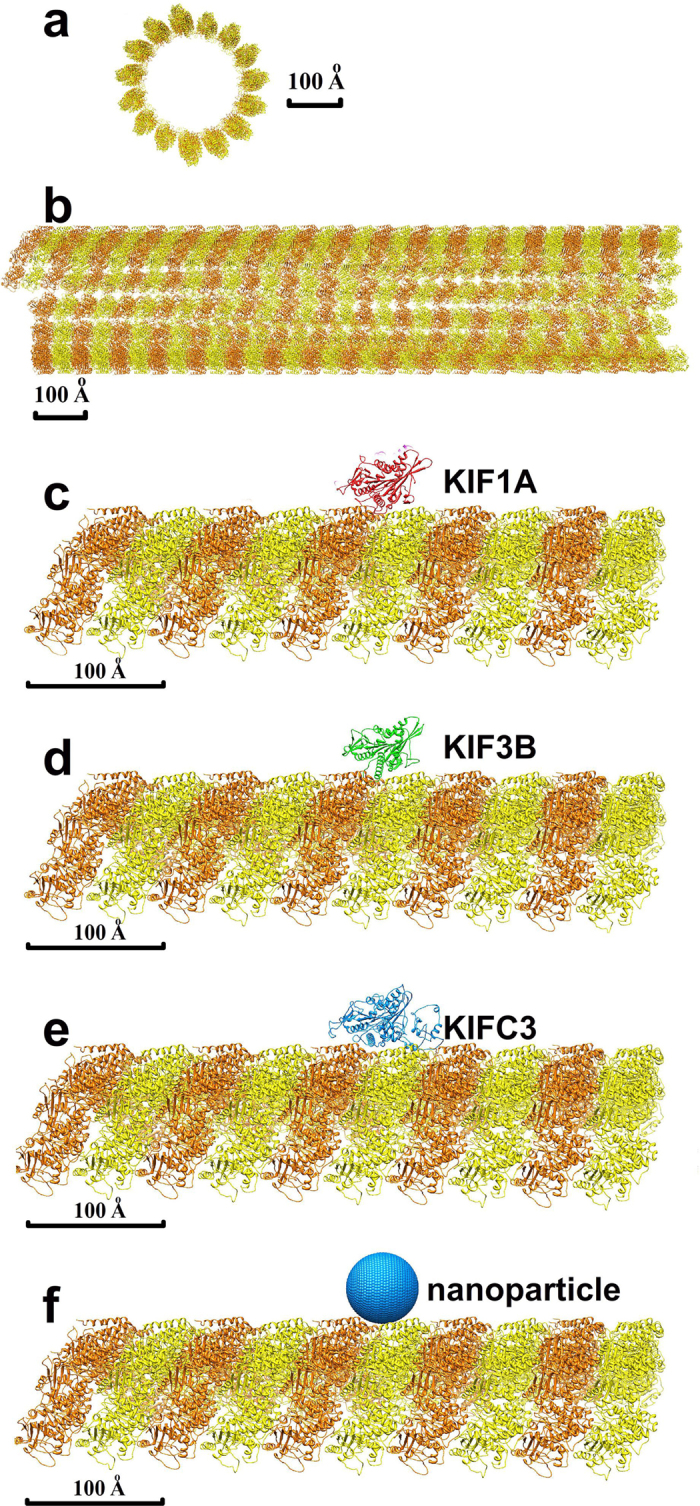
(**a**) The end view of the modeled microtubule. (**b**) The side view of the modeled microtubule. α-tubulin is orange and β-tubulin is yellow in both panels. The model was constructed using symmetry information from PDBID 3J2U, and rotation and translation manipulations were applied to the tubulins to model a 16-dimer length microtubule segment. **(c**–**f)** Details of the computational system. Each panel shows details of the specific ligand associated to the microtubule in which α-tubulin is orange and β-tubulin is yellow. (**c**) KIF1A, a kinesin-3, motor domain associated to a microtubule in its initial location of the simulation. (**d**) KIFC3, a kinesin-14, motor domain associated to a microtubule in initial location of the simulation. (**e**) Cin8, a bidirectional kinesin in its initial location of the simulation. (**f**) A uniformly charged nanoparticle associated to a microtubule in its initial location of the simulation.

We used this binding pathway, enabled by the computational focusing approach, to compare the electrostatic interactions between various kinesin motor domains and a complete 1280 Å long segment of a microtubule (Fig. 2). Specifically, we compared multiple N-kinesins, including kinesin-3 family member KIF1A, kinesin-2 family member KIF3B, a kinesin-14 family member of C-kinesin, KIFC3, and kinesin-5 family member Cin8, which has bi-directionality (Fig. 2). There are multiple differences between these motors, but of particular interest are the specific differences in both net charge (the Cin8, KIF1A, KIF3B, and KIFC3 motor domains we modeled have net charge of +15, +5, +4, and +9, respectively^50^) and charge distribution. We also compared the kinesins to a +32 charged nanoparticle (Fig. 2) as a uniformly distributed charge control. We started the pathway of each motor at the experimentally determined kinesin binding position on microtubule. Once the motor proceeded a distance of 80 Å from the starting position towards the plus- or minus-end of microtubule, which is the length of a tubulin dimer, the simulation stopped and a pathway was recorded. 1000 pathways were generated and analyzed for each case.

Each simulation took approximately 36,000 CPU hours (using Intel Xeon E5410 CPUs). We parallelized our computations on 200 CPUs, thus completing the simulation of 1000 pathways for each single motor in 180 hours, or just over a week. Had we made the same computations without using the focusing method, we estimate that it would have taken 200 CPUs multiple years to achieve the same level of computational accuracy.

### Electrostatic potential distribution around the microtubule shows potential valleys

**Figure 3:**
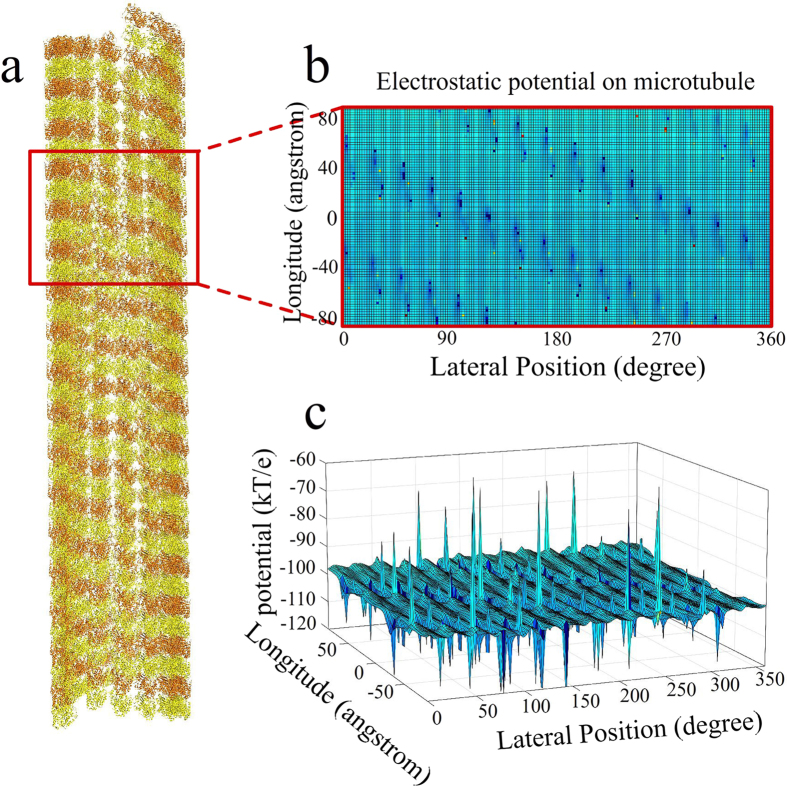
Electrostatic potential around microtubule calculated by DelPhi. (**a**) Structure of modeled microtubule showing a region of interest for the calculations. (**b**) Electrostatic potential around a segment of microtubule shows the periodic pattern of elongated electrostatic potential valleys along each protofilament of the microtubule. There are minimum electrostatic potential wells in each valley located at the experimentally determined kinesin motor domain binding locations. (**c**) Side view of the electrostatic potentials, periodically distributed potential valleys are found.

Electrostatic binding funnels can provide rough insight into the binding processes of a kinesin to a microtubule^51^,^52^,^53^. Thus, as a first step, before using the kinesin models, we calculated the electrostatic potential at probe points that we positioned 2 Å from the surface of microtubule using DelPhi’s “site potential” module. By plotting the electrostatic potential map, we see a periodic pattern of elongated electrostatic potential valleys along each protofilament of the microtubule (Fig. 3). There are minimum electrostatic potential wells in each valley, and the periodicity along the length is about 80 Å, corresponding to the length of a tubulin dimer (Fig. 3). Because kinesin motors carry positive net charges, the elongated negative electrostatic potential valleys suggest that the microtubule provides electrostatic forces that guide kinesin motors to walk along, rather than around, the microtubule.

### Kinesin motors and nanoparticles with uniform charge distribution diffuse along the length of microtubules and do not jump from protofilament to protofilament

We simulated binding pathways for the N-kinesins and C-kinesins (movies 1–4 for examples). We found that kinesin motors diffuse along the longitudinal direction of the microtubule in all generated pathways; no lateral diffusion was observed. This indicates the importance of electrostatic energy in directing kinesins to transport their cargo along the most direct, efficient path; electrostatic valleys (Fig. 3) do guide the kinesin motors along the length of microtubule.

**Figure 4:**
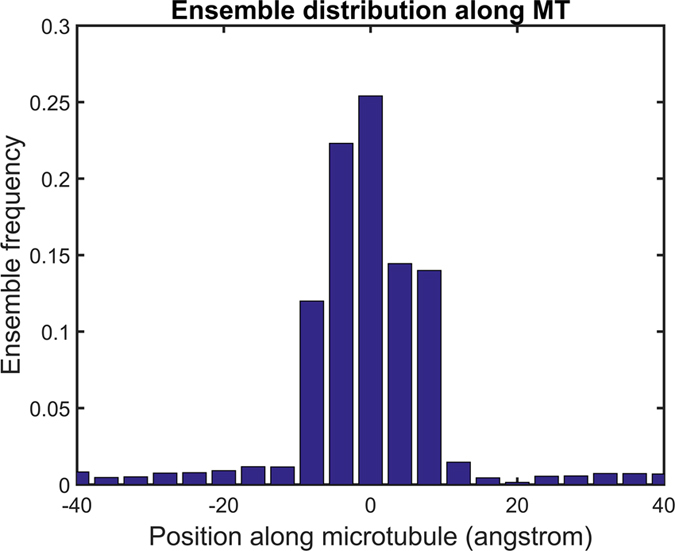
Ensemble distribution of kinesin motor along the microtubule. This data, from KIF1A, is typical for other motors, and it represents ensembles of structures from 1,000 simulations.

As kinesin motor domains diffuse long the microtubule, they are most frequently found at the experimentally determined binding positions on microtubules (Fig. 4). This indicates that electrostatic binding energy funnels exist around the binding sites, which we find to be approximately 15 Å long. Kinesin motors need energy to escape from these funnels. In the Monte Carlo simulations, the effect of such external energy is mimicked by carrying out the modeling at higher than physiological thermal energy (T = 500 K). In cells or *in vitro* experiments, motors likely need additional energy to drag them out of the binding energy funnel. Much experimental evidence suggests that the conformational changes of kinesin’s neck linker associated with the hydrolysis of ATP provide such a force^41^,^42^,^43^,^44^. Once this force pulls the motor out of the energy funnels, our calculations suggest that the motor domain moves to its next binding site with guidance from the electrostatic valleys in the energy landscape.

**Figure 5:**
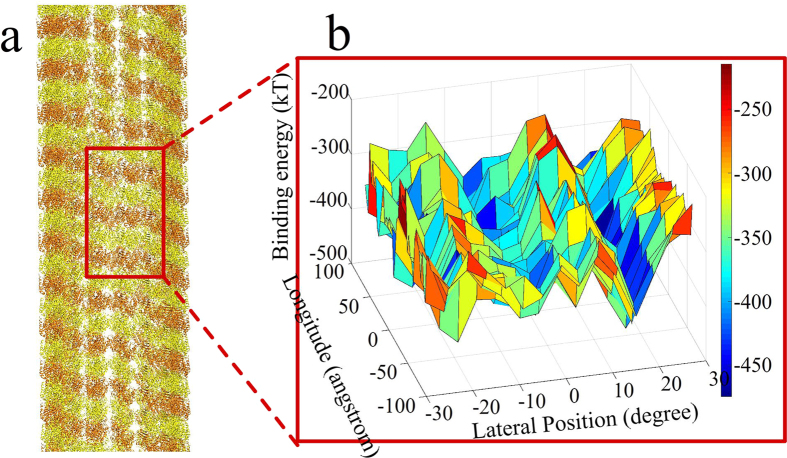
Electrostatic binding energy landscape of a nanoparticle and a microtubule. (**a**) Structure of modeled microtubule showing a region of interest for the calculations. (**b**) The binding energy landscape of a 3 dimer × 3 dimer segment of microtubule shows the periodic pattern of elongated nanoparticle binding energy valleys along each protofilament of the microtubule. There are binding energy wells in each valley located at the experimentally determined kinesin motor domain binding locations.

We also calculated the binding energy landscape of the nanoparticle to the microtubule. Similar to the electrostatic potential valleys seen with the test points (Fig. 3), we found binding energy valleys along the microtubule (Fig. 5) with the nanoparticle.

We simulated binding pathways for the nanoparticle (movie 4). We found that, like kinesin motors, nanoparticles diffuse along the longitudinal direction of the microtubule in all generated pathways; no lateral diffusion was observed. This corresponds to the observed behavior of nanoparticles diffusing along microtubules rather than around them^54^.

### The direction the calculated pathways correspond to the direction of kinesin motility

We further analyzed the Monte Carlo generated pathways to investigate the tendency of kinesin motor domains and nanoparticles to preferentially drift in particular directions along the microtubule. We found that the C-kinesin motors prefer to proceed to the minus-end of the microtubule while N-kinesin motors tend to prefer the plus-end (Table 1). Cin8 is a noted exception to the trend of N-kinesin motors preferring the plus-end. Cin8 is a unique N-kinesin that exhibits bi-directionality; single Cin8 motors walk to the minus-end of microtubules, but teams of Cin8 walk to the plus-end of microtubules^35^. In accordance with experimental observations^35^, we found that that electrostatic interactions guide the single Cin8 motor to the minus-end of microtubule (Table 1).

We found that the nanoparticle diffused to the plus-end with a probability of 0.53 +/−0.02 (Table 1), indicating that the electrostatic interaction doesn’t have any preference to directions along the microtubule for the nanoparticle. The non-preference has also been demonstrated in experiments^54^. This suggests that the specific charge distribution on kinesin motor domains is critical to the directional preference.

## Discussion

We developed a multiscale method to combine discrete sampling, Monte Carlo simulation, and a computationally focused energy calculation approach, which enables modeling of binding phenomena involving large biological objects. This multiscale method enabled us to study kinesin motor domain binding pathways along microtubules with desired accuracy, reducing the estimated computation time from multiple years down to just a week. We demonstrated the method’s ability to correctly predict the preferential directionality of various kinesin motor domains and that of a uniformly charged nanoparticle. The entire package can be downloaded from the URL: http://compbio.clemson.edu/downloadDir/main.tar.gz

### Electrostatic focusing enables the study of large-scale protein-protein interaction mechanisms

The multiscale method enabled us to calculate the binding energy landscape and likely pathways of various kinesins on complete microtubules with unparalleled accuracy and speed. Because of the scale of our simulation, we discovered evidence of how electrostatic forces play an important role in two key aspects of kinesin motility. First, we showed that simulated kinesin motor domains do not switch protofilaments as they move, suggesting that electrostatic forces are a possible mechanism for the well-known phenomenon of kinesins walking parallel to, not around, microtubules^55^,^56^. Second, we showed that the simulated motility of N-kinesins tend to be plus-end directed while C-kinesins tend to be minus-end directed, suggesting that specific electrostatic charge distribution on kinesin motor domains biases the motion in a polarized manner. Together, our results reveal long-range contributions of electrostatic potential to motor motility, beyond guiding and aligning kinesin motor domains to their binding locations on tubulin dimers through a binding funnel^45^.

The multiscale method leveraged previous efforts to simulate large protein-protein systems in a computationally efficient way^11^,^12^,^13^,^16^, enabling a step-function increase in the size of systems for which computation is tractable. This method enabled us to observe larger-scale electrostatic force effects than previously reported for microtubule-kinesin systems^45^. The results demonstrate that our methodology is particularly suitable for modeling electrostatic potential and binding energy in large systems without obvious charge symmetry. Such systems include microtubule dynamics^11^, mitochondrial complex^10^, photosynthetic complex^43^ and others^1^,^2^. In such systems, without charge or geometrical symmetry, taking into account long-range electrostatic effects will influence not only the amplitude of calculated electrostatic potential, but also its 3D profile.

### Enhancements to the computational focusing method enhance its potential impact

The current implementation of the multiscale method requires computation of the binding energy at each sampling position, as determined by the sampling module. In the simulations of kinesin motors on microtubules we present here, this is 36,000 total, unique binding energy calculations. However, we find that, in all 1,000 pathways, only 9% of these sampling positions were ever occupied by the motor domains. Therefore, it is theoretically possible to gain another nearly 10-fold decrease in computation time by avoiding computations of inaccessible states.

The multiscale method can be applied in conjunction with linear or non-linear Poisson-Boltzmann. Additional energy terms can easily be appended to the energy module to reflect the specificity of the modeled system. The discretization of the sampling space is also adjustable as well as the volume of the space to be sampled. This further extends the applicability of the approach to various and diverse systems.

### Additional considerations to improve modeling protocol

The current version of the multiscale protocol considers the large host (receptor) and the small binding protein (ligand) to be rigid bodies, both structurally and with unchangeable charge distribution. However, as the kinesin binding domain samples different positions and orientations around the microtubule, it and the microtubule may undergo conformational and ionization changes. Such phenomena are well documented for protein-protein interactions^57^,^58^,^59^,^60^,^61^. To be incorporated in the current protocol, one should explore conformational space (or have pre-determined conformational ensemble of structures) and carry out pKa calculations at each Monte Carlo step. In addition, if such changes are generated, the corresponding energies must be re-calculated including taking into account the changes of the internal energy of both the receptor and the ligand. This will make the computational protocol extremely time demanding.

## Methods

### DelPhi Finite Difference Poisson-Boltzmann Solver

In our approach we utilize DelPhi^48^,^49^,^62^, which solves the Poisson-Boltzmann equation to obtain the electrostatic potential distribution and electrostatic forces at desired positions in and around biological macromolecules. It uses structural PDB files as its primary input, and its results can be viewed in standard molecular viewers. DelPhi implements the finite different method to solve the Poisson-Boltzmann Equation on a set of grids. A resolution of 2 grids/Å is sufficient for DelPhi to make accurate calculations. DelPhi has been parallelized in an efficient way, which makes it capable of calculating the electrostatic potential in large structures, such as viruses, using multiple CPUs^10^,^48^.

### Focusing method

We developed a new focusing method to accelerate the electrostatic calculations. When a system is as large as 1000 Å, the mesh consists of 2,000 × 2,000 × 2,000 grid points, which requires a large amount RAM (more than 100 GB) and takes long time to complete the calculations. However, in many systems, the area of concern is usually only part of the system. Our focusing method exploits this fact to accelerate electrostatic energy calculations.

**Figure 6:**
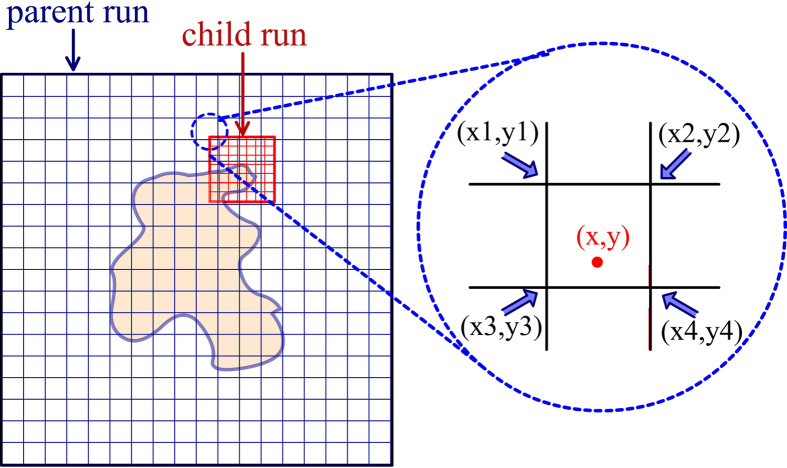
A two-dimensional representative example system with a coarse grid indicating the low-resolution nature of the electrostatic calculations done in a parent run. The area of interest and a representative grid indicating the high-resolution nature of the electrostatic calculations done in the child run is shown in red. The detail shows the boundary of the area of interest upon which the electrostatic potential calculated in the parent run is applied as a boundary condition.

Our focusing method enables DelPhi to focus on the area of interest while accounting for the entire large system as an environment by using parent-child runs. In the parent run, DelPhi calculates and saves the electrostatic potential of the entire system at low resolution, phimap0. In subsequent child runs, DelPhi calculates the potential in the area of interest at a higher resolution. Child runs read the electrostatic potential from the parent run (phimap0) and project that potential onto the boundary of the area of interest. Then DelPhi solves the Poisson-Boltzmann Equation in the child run and creates a high-resolution electrostatic potential, phimap1 (Fig. 6). The implementation of focusing method allows the area of interest to be located anywhere within the entire system and to have any resolution.

This focusing method significantly saves calculation time, because: 1) only the region of interest is calculated at high resolution, while the rest of the system is modeled at low resolution; 2) multiple child runs share the same parent run; and 3) for very large simulations, the focusing method can be applied in multiple levels.

### Sampling module

This module generates an ensemble of structures. The large object (receptor) is fixed in space and the small molecule (ligand) is translated and rotated with respect to the large object using translation and rotation matrices. Both the large object and the small molecules are treated as rigid bodies. The ligand position and orientation is stored in a 6 dimensional vector (x, y, z, *ϕ*, *θ*, *Ψ)*, where x, y, z are the Cartesian space coordinates of the mass center of ligand and *ϕ*, *θ*, *Ψ* are the Euler angles that determine the orientation of the ligand.

**Figure 7:**
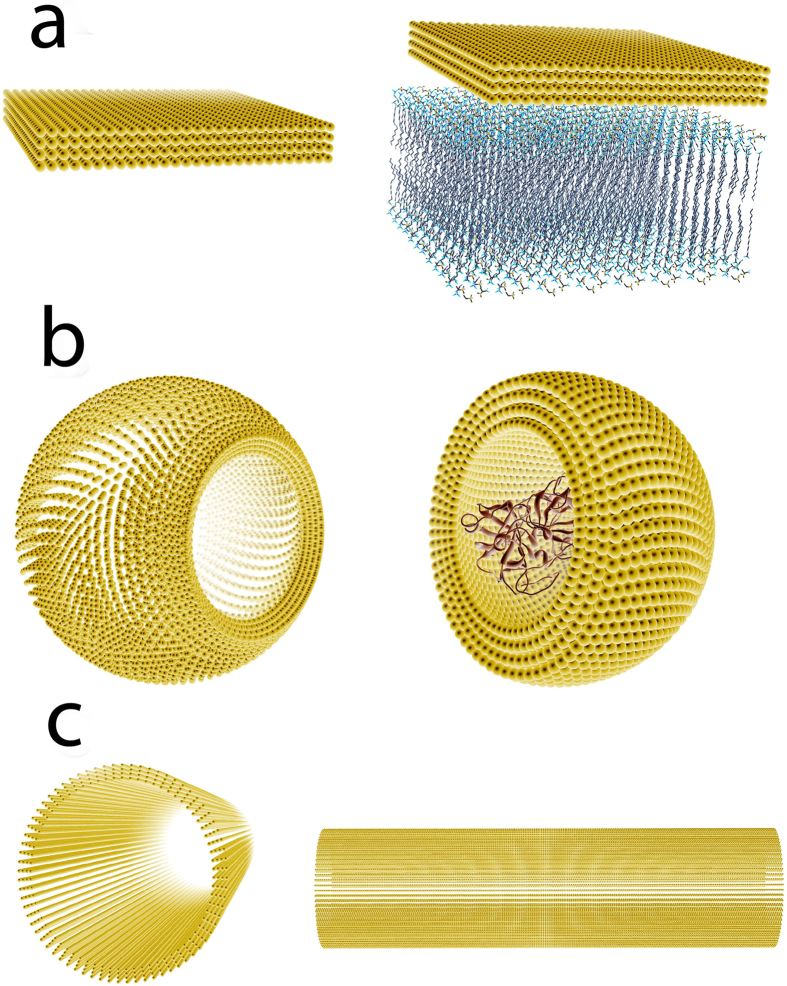
Illustrations of the rigid-body sampling module. (**a**) Cuboidal sampling is used for energy calculations between a ligand and a planar receptor, e.g. a membrane generated by ProBLM Web-server^73^ is shown. (**b**) Spherical sampling is used for energy calculations between a ligand and a globular protein receptor, e.g. a barnase [PDBID: 1brs] is shown. (**c**) Cylindrical sampling is used for energy calculations between a ligand and a cylindrical receptor. In each panel, the yellow dots represent mass centers of rotamers of the ligand.

The sampling module provides three sampling strategy options (Fig. 7): cuboidal, spherical, and cylindrical samplings. The cuboidal sampling option distributes the mass center of the ligand in a cuboidal shape (Fig. 7a) with respect to the receptor, and then it generates rotamers at each mass center by varying the rotation vector *ϕ*, *θ*, *Ψ*. The cuboidal sampling method is designed for positioning a ligand near a flat larger object, e.g. a G protein binding to a GPCR on a membrane^2^. The spherical sampling option distributes the mass center of the ligand in a spherical or spherical shell shape (Fig. 7b) with respect to the receptor, and then generates rotamers at each mass center. The spherical sampling module is designed for systems with spherical geometry, e.g. an antibody binding to large virus. The cylindrical sampling option distributes the mass center of the ligand in a cylindrical or a hollow cylindrical shape (Fig. 7c) with respect to the receptor, and then generates rotamers for each mass center. The cylindrical sampling method is designed for systems with cylindrical geometry, e.g. proteins binding to DNA or kinesins walking on a microtubule.

### Energy module

The energy module calculates the total energy, *E*_*total*_, for each structure generated by the sampling module. The total energy is comprised of three energy terms:


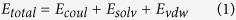


where *E*_*coul*_ is the Coulombic (electrostatic) energy, *E*_*solv*_ is the solvation energy, and *E*_*vdw*_ is the Van der Waals energy. The electrostatic and solvation energies are calculated by DelPhi by solving Poisson-Boltzmann equation, with the AMBER force field^63^, and at a 150 mM salt concentration. The electrostatic energy is calculated using the computational focusing method, detailed above. The Van der Waals energy is calculated in the energy module, using the Lennard-Jones potential for each pair of atoms *i* and *j*


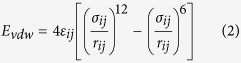


where *ε*_*ij*_ is the potential well depth, *σ*_*ij*_ is the characteristic distance parameter, and *r*_*ij*_ is the distance between atoms *i* and *j*. *ε*_*ij*_ and *σ*_*ij*_ depend on the atom types, and they are obtained from the AMBER force field. The total binding energy is calculated for each pre-generated conformation of the ligand and stored in a lookup table.

There are cases in which the rigid body sampling method generates structures that contain two atoms that are extremely close together, causing the Coulombic energy and Van der Waals energy to become infinite or unrealistically large. A “soft” Coulombic and Van der Waals approach is implemented to avoid extreme values and tolerate small clashes: when the distance between two atoms is less than 1 Å, the energy module resets the distance to 1 Å. This is similar to other soft Van der Waals energy approaches, which have been successful in solving rigid body protein-protein docking problems^46^,^64^,^65^.

### Pathway generation module

The Monte Carlo simulation generates plausible pathways of the ligand based on Metropolis algorithm and using the binding energies in the lookup table. At the beginning of the simulation, the ligand is located at a starting position, S_0_(x_0_, y_0_, z_*0*_, *ϕ*_0_*, θ*_0_*, Ψ*_0_), as specified by the user. The pathway module then generates new positions of the small molecule in stepwise manner. At the n^th^ step of simulation with position S*n*(x_*n*_, y_*n*_, z_*n*_, *ϕ*_*n*_, *θ*_*n*_, *Ψn*), the pathway module generates a random vector (dx, dy, dz, d*ϕ*, *dθ*, *dΨ)* to determine the next “attempted” position of the small molecule: S′_*n*+1_ (x_*n*+1_, y_*n*+1_, z_n+1_, *ϕ*_*n*+1_, *Ψ*_*n*+1_), where (x_*n*+1_, y_*n*+1_, z_n+1_, *ϕ*_*n*+1_, *Ψ*_*n*+1_) = (x_*n*_ + dx, y_n_ + dy, z_n_ +dz, *ϕ*_*n*_ + *dϕ*, *θ*_*n*_ + *dθ*, *Ψ*_*n*_ +d*Ψ)*. The Metropolis algorithm is used to decide if the attempted position S′_*n*+1_(x_*n*+1_, y_*n*+1_, z_n+1_, *ϕ*_*n*+1_, *θ*_n+1_, *Ψ*_*n*+1_) is accepted. If Δ*E*(*n* +1, *n*), the energy difference, between S′_*n*+1_(x_*n*+1_, y_*n*+1_, z_n+1_, *ϕ*_*n*+1_, *θ*_n+1_, *Ψ*_*n*+1_) and S_*n*_(x_*n*_, y_*n*_, z_*n*_, *ϕ*_*n*_, *θ*_*n*_, *Ψ*_*n*_), is less than 0, then the attempted structure is accepted. If Δ*E*(*n* +1, *n*) is greater than 0, there is still a probability, *ρ*_*n*_, of accepting the attempted structure. *ρ*_*n*_ is determined by Boltzmann statistics


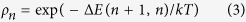


where k is Boltzmann’s constant and T is the temperature that attempted structure is accepted. When a position is accepted, then S_*n*+1_(x_*n*+1_, y_*n*+1_, z_*n+*1_, *ϕ*_*n*+1_, *θ*_*n+1*_, *Ψ*_*n*+1_) = S′_*n*+1_(x_*n*+1_, y_*n*+1_, z_*n+*1_, *ϕ*_*n*+1_, *θ*_*n+1*_, *Ψ*_*n*+1_). Alternatively, when the attempted structure is rejected, with probability 1−*ρ*_*n*_, the ligand stays at the previous state and S_*n*+1_(x_*n*+1_, y_*n*+1_, z_*n+*1_, *ϕ*_*n*+1_, *θ*_*n+1*_, *Ψ*_*n*+1_) = S_*n*_(x_*n*_, y_*n*_, z_*n*_, *ϕ*_*n*_, *θ*_*n*_, *Ψ*_*n*_).

### Microtubule, kinesin motors and nanoparticles

The microtubule is modeled based on cryo-EM structure of bovine tubulin (PDBID: 3J2U^66^, Figs 2 and 3), in which the high resolution crystal structure of tubulin (PDBID: 1JFF)^67^ is fitted. Based on the rotation and translation information in 3J2U, a piece of microtubule is modeled with the length of 1280 Å, and the diameter is 300 Å (Fig. 2).

We used the following N-kinesin motor domains. The Cin8 motor domain is an ADP-associated, *S. cerevisiae* kinesin-5 structure modeled by SWISS-MODEL^68^ using the template of human Eg5, PDBID 1II6^69^. Note that the sequence similarity between Eg5 and Cin8 is 59%. The KIF1A motor domain is an AMPPNP-associated mouse kinesin-3 PDBID: 1VFV^70^. The KIF3B motor domain is an ADP-associated human kinesin-2, PDBID 3B6U^71^.

We used the following C-kinesin motor domain. The KIFC3 motor domain is an ADP-associated human kinesin-14, PDBID: 2H58^71^.

We used a nanoparticle as a uniform charge control. We generated it by ProNOI^72^ with a uniform net charge of +32, which is similar to the net charge of nanoparticles studied experimentally^54^.

Cylindrical sampling method is used for each simulation. The sampling module samples the structures of each kinesin or nanoparticle from -88 to 88 Å along the longitude direction, which covers more than the length of two tubulin dimers; the perpendicular distance between kinesin (or nanoparticle) and microtubule surface is from 0 to 20 Å; the lateral range is 56 degrees, which covers more than the width of two protofilaments. The range of the sampling is large enough that, in our simulations, no kinesins or nanoparticles reach the edge of the sampling range. The resolution of the distribution is 4 Å. *In vivo*, due to the binding of cargos, the kinesin head is not totally free to rotate. Therefore, in our simulation, at each position, 9 rotomers are generated to model the orientations of kinesins. For the nanoparticle, rotamers are not generated due to its symmetry sphere shape.

All of these structures including kinesin motors and nanoparticle are available online:

http://figshare.com/articles/data_for_kinesin_motor_simulation/1613452.

## Additional Information

**How to cite this article**: Li, L. *et al.* Multiscale method for modeling binding phenomena involving large objects: application to kinesin motor domains motion along microtubules. *Sci. Rep.* **6**, 23249; doi: 10.1038/srep23249 (2016).

## Supplementary Material

Supplementary Information

Supplementary movie 1

Supplementary movie 2

Supplementary movie 3

Supplementary movie 4

## Figures and Tables

**Table 1:** Kinesin’s directional probability.

PDB	Motor	Family	Type	Probability to plus end
1vfv	KIF1A	kinesin-3	N-kinesin	0.86
3b6u	KIF3B	kinesin-2	N-kinesin	0.86
2 h58	KIFC3	kinesin-14	C-kinesin	0.18
	Cin8	kinesin-5	N-kinesin	0.19
			nanoparticle	0.53
