# Supplementary material for: Multiscale method for modeling binding phenomena involving large objects: application to kinesin motor domains motion along microtubules
Source: Sci Rep. 2016 Mar 18;6:23249. doi: 10.1038/srep23249 (PMC4796874; doi:10.1038/srep23249)
Supplement: Supplementary Information [file srep23249-s1.pdf]

Supplementary Information

Title:

Multiscale method for modeling binding phenomena involving large objects: application to kinesin motor domains motion along microtubules

Authors:

Lin Li, Joshua Alper, Emil Alexov*

Video captions:

Movie 1: Monte Carlo pathway for an N-kinesin motor (PDBID: 1vfv) along the microtubule.

Movie 2: Monte Carlo pathway for a C-kinesin motor (PDBID: 2h58) along the microtubule.

Movie 3: Monte Carlo pathway for Cin8 motor along the microtubule.

Movie 4: Monte Carlo pathway for a nanoparticle along the microtubule.
